# Frailty Recovery Following Minimally Invasive Surgery: An Emerging Perspective on Surgical Benefits in Elderly Colorectal Cancer Patients

**DOI:** 10.1002/ags3.70070

**Published:** 2025-08-03

**Authors:** Hajime Ushigome, Yushi Yamakawa, Shunsuke Hayakawa, Akira Kato, Takuya Suzuki, Takafumi Sato, Hiroyuki Sagawa, Ryo Ogawa, Hiroki Takahashi, Shuji Takiguchi

**Affiliations:** ^1^ Department of Gastroenterological Surgery Nagoya City University Graduate School of Medical Sciences Nagoya Japan

**Keywords:** colorectal cancer, frailty, frailty recovery, minimally invasive surgery, robot‐assisted surgery

## Abstract

**Background:**

Frailty is common among elderly colorectal cancer (CRC) patients and affects both perioperative and long‐term outcomes. However, many aspects of how minimally invasive surgery (MIS) influences frailty remain unclear. Moreover, very few reports have specifically evaluated postoperative changes in frailty status.

**Methods:**

In this prospective observational study, 239 CRC patients aged ≥ 70 years undergoing MIS with R0 resection were assessed for frailty using the FRAIL Scale and Kihon Checklist preoperatively and 1 year postoperatively. Short‐ and long‐term oncological outcomes and frailty status changes were analyzed.

**Results:**

A total of 88 patients (37%) were diagnosed as frail on the basis of either of the frailty assessment tools, and MIS was performed in all cases. Short‐term outcomes were comparable between frail and non‐frail patients; however, overall survival (OS) was significantly worse in frail patients (log‐rank, *p* = 0.01). Among the 141 patients who remained recurrence‐free for 1 year and completed the second questionnaire, frailty status significantly improved according to the FRAIL Scale (*p* = 0.001). In patients whose frailty status improved, significant improvements in hemoglobin levels, the prognostic nutritional index, and psoas muscle index were observed compared with their preoperative values.

**Conclusions:**

Curative MIS was safely performed in elderly frail colorectal cancer patients without increasing perioperative complications; however, their long‐term outcomes remained poor. Nevertheless, in patients who remained recurrence‐free, frailty status significantly improved 1 year after surgery, suggesting an emerging potential benefit of surgical resection in the elderly population.

## Graphical abstract

This prospective study investigated the impact of MIS on frailty and outcomes in elderly CRC patients. While frail patients showed lower OS, MIS was safely performed. Among recurrence‐free patients, frailty status improved at 1 year, suggesting that curative surgery may offer not only oncologic benefits but also potential for frailty improvement.

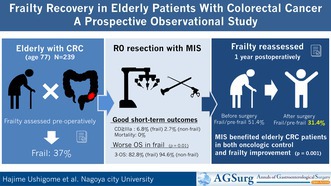

## 1 Introduction

Colorectal cancer (CRC) is one of the most common gastrointestinal malignancies in both Asia and Western countries. In recent years, the aging of the global population has led to a steady increase in elderly patients with CRC, with approximately 50% of patients aged 70 years or older and more than 40% aged 75 years or older [1].

Surgical resection remains the cornerstone of treatment for CRC in elderly patients; however, age‐related vulnerabilities—physical, psychological, and social, collectively referred to as frailty—are frequently observed in this population. Frailty has been identified not only as a risk factor for poor short‐term outcomes but also as a predictor of long‐term prognosis, functional recovery, and quality of life [2, 3, 4, 5, 6]. Therefore, frailty assessment, which evaluates the overall vulnerability of elderly patients beyond conventional indicators such as age and the presence of comorbidities, is crucial in the management of elderly patients with CRC [5, 6, 7].

Minimally invasive surgery (MIS) has become widely adopted for CRC due to its association with reduced postoperative morbidity and faster recovery compared with open surgery [8, 9]. Although short‐term outcomes of MIS, even in frail patients, have been relatively well documented [10, 11, 12, 13], the overall evidence remains limited, and data on long‐term outcomes are still scarce. Moreover, research focusing specifically on postoperative changes in frailty status remains insufficient, despite its potential relevance to optimizing postoperative care and improving survivorship in elderly patients.

A variety of tools are available for assessing frailty, but it is recommended that clinicians use validated and reliable instruments tailored to their specific clinical or research settings [5, 14]. The FRAIL scale is one such internationally recognized screening tool for physical frailty, consisting of five components: fatigue, resistance, ambulation, illnesses, and weight loss. It is simple and feasible for bedside use [5, 14, 15, 16]. The Kihon Checklist (KCL), developed by the Japanese Ministry of Health, Labour and Welfare, consists of 25 self‐reported items evaluating multiple domains including physical function, cognitive function, social participation, nutritional status, and psychological condition. KCL comprehensively covers all components of the geriatric assessment and has been widely applied in clinical and research settings in Japan [14, 15, 17, 18].

The aim of this study is to investigate short‐ and long‐term oncological outcomes, as well as changes in frailty status 1 year after MIS in elderly patients with CRC, using standardized frailty assessment tools.

## 2 Materials and Methods

### 2.1 Study Design

This prospective observational study was conducted using a questionnaire survey designed by the Nagoya City University Graduate School of Medical Sciences. Patients scheduled to undergo primary CRC resection were routinely assessed for frailty preoperatively and 1 year postoperatively using two validated questionnaires. Prospectively collected questionnaire data were retrospectively analyzed.

### 2.2 Questionnaires

Frailty was assessed using two types of questionnaires: the FRAIL Scale and the Kihon Checklist. The FRAIL Scale is classified as a rapid screening tool for physical frailty. It consists of five questions, scored from 0 to 5. Based on the total score, patients were categorized as non‐frail (0 points), pre‐frail (1–2 points), or frail (3–5 points) (Table S1).

The Kihon Checklist is categorized as a metrics‐computed assessment tool evaluating physical, psychological, and social frailty. It includes 25 questions, scored from 0 to 25 points. Based on the total score, patients were classified as non‐frail (0–3 points), pre‐frail (4–7 points), or frail (≥ 8 points) (Table S2).

### 2.3 Patient Population

From November 2019 to August 2023, 685 patients underwent primary CRC resection at Nagoya City University. Patients were excluded for the following reasons: age under 70 years (*n* = 262), stage IV disease (*n* = 59), open surgery (*n* = 5), presence of active malignancies under treatment (*n* = 29), R1 or R2 resection status (*n* = 11), or missing questionnaire data due to emergency surgery or patient refusal or missing data (*n* = 80). After these 446 patients were excluded, 239 patients were included in the analysis of the first questionnaire.

**FIGURE 1:**
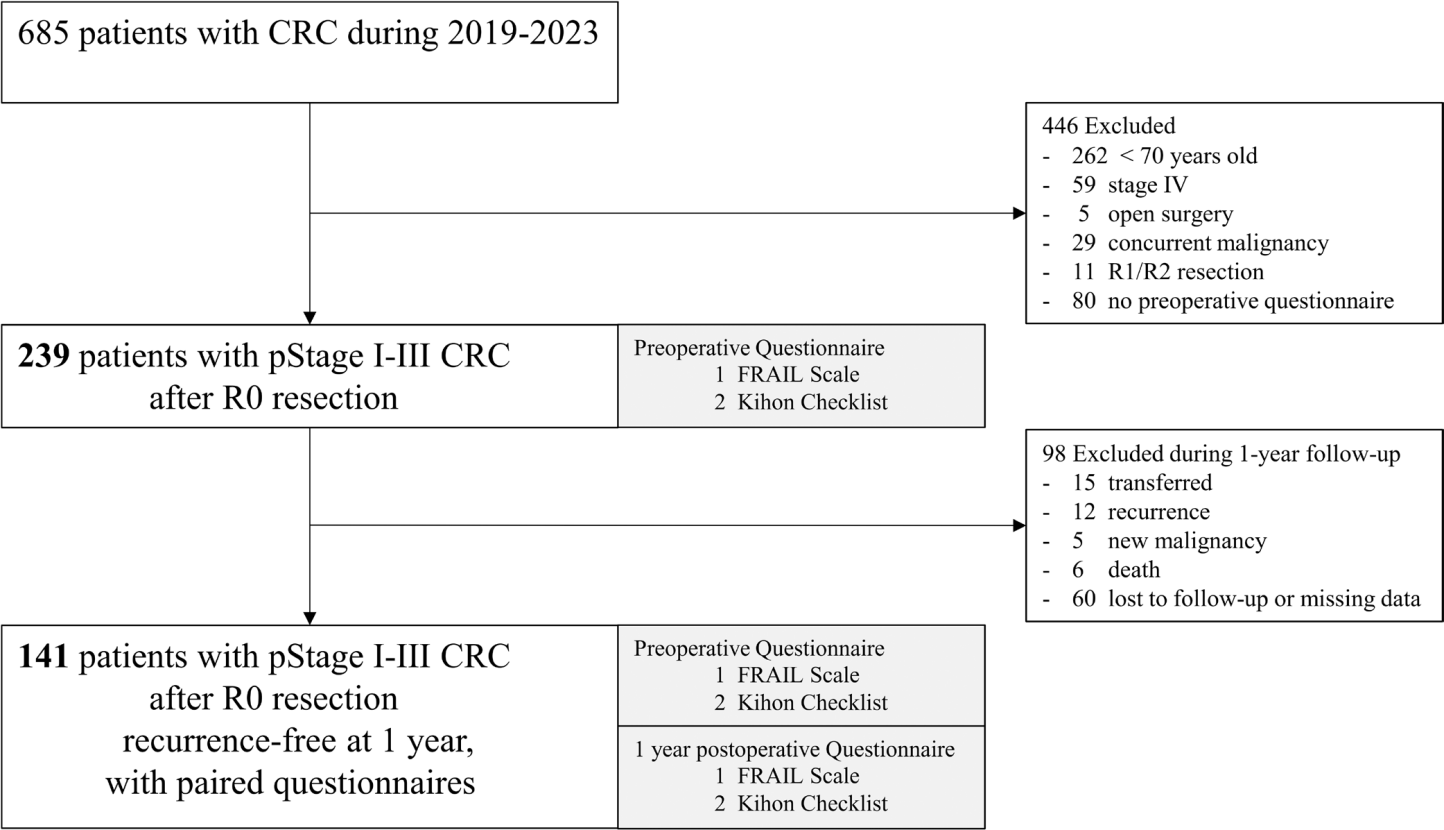
Flow diagram for patient selection in colorectal cancer study.

Among the 239 patients, some were later excluded because of transfer to other hospitals (*n* = 15); disease recurrence (*n* = 12); development of other malignancies (*n* = 5); death (*n* = 6); or loss to follow‐up, refusal, or missing data (*n* = 60). Consequently, 141 patients were included in the analysis of the second questionnaire (Figure 1). Patient background was assessed using standard clinical variables as well as the American Society of Anesthesiologists physical status (ASA‐PS) [19], the Charlson Comorbidity Index (CCI) [20], the Prognostic Nutritional Index (PNI) [21], and the Psoas Muscle Index (PMI) [22, 23].

As part of the evaluation of short‐term postoperative outcomes, we assessed whether total mesorectal excision (TME), tumor‐specific mesorectal excision (TSME) [24] (hereafter collectively referred to as TME), and complete mesocolic excision (CME) [25] were successfully performed. Procedures were defined as “complete” if dissection along the embryological planes was achieved. For CME, high ligation of the feeding vessels at the root of the corresponding artery was also required. Although high ligation is not a standard component of TME or TSME definitions, it was used here specifically to define the completeness of CME. The completeness of these procedures was assessed intraoperatively by the operating surgeon based on standard anatomical criteria.

### 2.4 Statistical Analysis

#### 2.4.1 Quantitative Data Are Expressed as Medians and Ranges

1. Differences in patient characteristics and short‐term outcomes between the frail and non‐frail groups among patients who completed the preoperative questionnaire were analyzed using the Wilcoxon rank‐sum test and the χ^2^ test. Overall survival (OS) and relapse‐free survival (RFS) in the two groups were analyzed using the Kaplan–Meier method and compared with the log‐rank test.
2. Among patients who completed both preoperative and 1‐year postoperative questionnaires, changes in frailty and pre‐frailty status were evaluated using the McNemar test. Changes in various variables from the preoperative to postoperative periods within the pre‐frail/frail and non‐frail groups were assessed using the Wilcoxon signed‐rank test and the McNemar test, as appropriate. Differences in variable changes between the pre‐frail/frail and non‐frail groups were analyzed using repeated measures analysis of variance (ANOVA).

A *p*‐value of < 0.05 was considered statistically significant. All statistical analyses were performed using JMP software, version 8.0.2 (SAS Institute, Cary, NC, USA).

## 3 Results

The median age of the 239 patients who completed both preoperative questionnaires was 77 years. Among them, 59% had colon cancer and 41% had rectal cancer. Based on the psoas muscle index (PMI), 63% were diagnosed with low PMI.

Eighty‐eight patients (37%) were diagnosed as frail by either the FRAIL Scale or the Kihon Checklist. These patients were significantly older and more likely to be female compared with the non‐frail group. In addition, frail patients tended to have worse performance status (PS), higher ASA‐PS classifications, and poorer nutritional assessments than non‐frail patients. Although the CCI tended to be higher in the frail group, the difference was not statistically significant (Table 1).

**TABLE 1:** Demographic and clinical features of the study population.

Variables, N0. (%) or Median (range)	All patients	Frail	Non frail	*p*
	(*n* = 239)	(*n* = 88)	(*n* = 151)	
Age (years)	77 (70–95)	80 (70–95)	75 (70–89)	0.001*
Male/Female	128: 111	37:51	91:60	0.007*
BMI (kg/m^2^)	22.1 (14.2–35.4)	21.8 (14.1–32.9)	22.3 (15.3–35.4)	0.687
Tumor location				
Colon	139 (59.1)	57 (64.7)	82 (54.3)	0.114
Rectum	100 (40.9)	31 (35.2)	69 (45.7)	
Obstruction				
Yes	32 (13.3)	13 (14.7)	19 (12.6)	0.632
No	207 (86.7)	75 (85.3)	132 (87.4)	
Performance status				
0–1	216 (90.3)	69 (78.4)	147 (97.4)	0.001*
2–4	23 (9.7)	19 (21.6)	4 (2.6)	
ASA‐PS				
1–2	187 (78.2)	62 (70.5)	125 (82.8)	0.023*
3–4	52 (21.8)	26 (29.5)	26 (17.2)	
Charlson comorbidity index				
0–2	203 (84.9)	71 (80.7)	132 (87.4)	0.369
3–4	30 (12.6)	14 (15.9)	16 (10.6)	
5 ≦	6 (2.5)	3 (3.4)	3 (2.0)	
Prognostic Nutritional Index	47 (29–68)	44.5 (30–58)	48.5 (29–68)	0.001*
Psoas Muscle Index	4.56 (1.8–10.9)	4.3 (1.8–7.7)	4.9 (2.2–10.9)	0.001*
Low PMI				
Yes	146 (63.4)	57 (67.9)	89 (60.9)	0.366
No	84 (36.6)	27 (32.1)	57 (39.1)	
Preoperative chemotherapy				
Yes	5 (2.1)	2 (2.3)	3 (1.9)	0.881
No	234 (97.9)	86 (97.7)	148 (98.1)	
Preoperative radiotherapy				
Yes	5 (2.1)	2 (2.3)	3 (1.9)	0.881
No	234 (97.9)	86 (97.7)	148 (98.1)	

Abbreviations: ASA‐PS, American Society of Anesthesiologists Physical Status Classification System; BMI, Body Mass Index; PMI, Psoas Muscle Index.

* Statistically significant (*p* < 0.05). PMI data were missing for 9 patients due to unavailability of preoperative imaging.

Table 2 summarizes short‐term outcomes. The non‐frail group tended to have a higher rate of complete TME or CME excision compared with the frail group (*p* = 0.122). The rate of incomplete TME or CME was 12.4% (11 cases) in the frail group and 6.6% (10 cases) in the non‐frail group, with no statistically significant difference between the groups. Among the cases with incomplete resection, the proportion of patients with stage II or higher advanced cancer was 64% in the frail group and 30% in the non‐frail group. In these cases, the incomplete resection was attributed to the selection of a safer surgical approach, taking into consideration the patients' age and comorbidities.

**TABLE 2:** Short‐term surgical and postoperative outcomes by frailty status.

Variables, N0.(%) or Median (range)	Frail	Non frail	*p*
	(*n* = 88)	(*n* = 151)	
Surgical approach			
Laparoscopic	42 (47.7)	87 (57.6)	0.139
Robotic	46 (52.3)	64 (42.4)	
Operation time	240 (94–750)	247 (117–882)	0.648
Bleeding	29 (0–680)	29 (0–1949)	0.761
TME or CME			
Complete	77 (87.5)	141 (93.4)	0.122
Not complete	11 (12.4)	10 (6.6)	
Pathological stage (%)			
0	3 (3.4)	2 (1.3)	0.187
I	18 (20.5)	48 (31.8)	
II	41 (46.6)	57 (37.8)	
III	26 (29.6)	44 (29.1)	
Morbidity (CD)			
0	67 (76.1)	124 (82.1)	0.263
I–II	15 (17.0)	23 (15.2)	
≧ III	6 (6.8)	4 (2.7)	
Mortality	0	0	
Postoperative in‐hospital stay	11 (6–105)	10 (6–92)	0.030*
Adjuvant chemotherapy	11 (12.5)	31 (20.5)	0.116
Oxaliplatin based	2 (18.2)	18 (58.6)	0.023*
Others	9 (81.8)	13 (41.9)	

*Note:* The distribution of oxaliplatin‐based and other regimens was analyzed only among patients who received adjuvant chemotherapy.

Abbreviations: CD, Clavien Dindo classification; CME, complete mesocolic excision; TME, total mesorectal excision (including TSME, tumor‐specific mesorectal excision).

* Statistically significant difference (*p* < 0.05).

There was no significant difference in postoperative complication rates between the two groups; however, the length of postoperative hospital stay was significantly longer in the frail group (*p* = 0.030).

Adjuvant chemotherapy was more frequently administered in the non‐frail group, and regimens containing oxaliplatin were significantly more common in the non‐frail group (*p* = 0.023).

**FIGURE 2:**
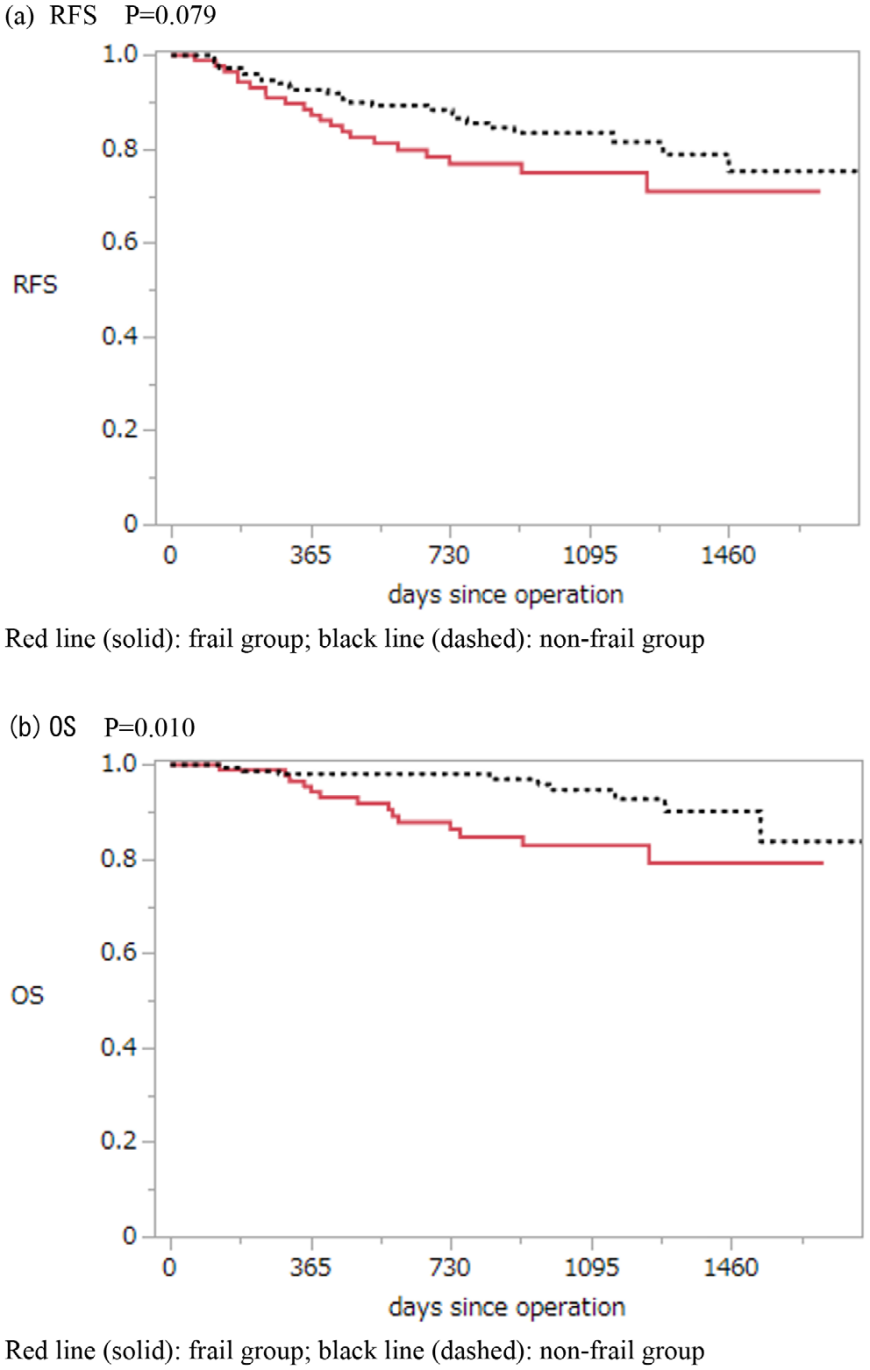
Kaplan–Meier curves for relapse‐free survival (RFS) and overall survival (OS) according to frailty status.

Regarding long‐term outcomes in the 239 patients who completed the preoperative questionnaire, the 3‐year RFS rates were 83.4% in the non‐frail group and 74.9% in the frail group, with no significant difference according to the log‐rank test (*p* = 0.078). In contrast, the estimated 3‐year OS rates were 94.6% and 82.8%, respectively, showing a significant difference between the groups (*p* = 0.010, log‐rank test) (Figure 2). In the frail group, 14 patients died, with cancer‐related deaths accounting for 7 cases (50%). In the non‐frail group, 10 patients died, and 4 deaths (40%) were cancer‐related. The median follow‐up period was 33.4 months.

Table 3 compares the results of 141 patients who completed both the preoperative and 1‐year postoperative questionnaires. Although the number of patients diagnosed as frail decreased in both the FRAIL Scale and the Kihon Checklist assessments, the differences were not statistically significant. However, when evaluating the number of patients classified as either frail or pre‐frail, a significant reduction was observed in the FRAIL Scale results 1 year postoperatively (*p* = 0.001).

**TABLE 3:** Changes in frailty status assessed by FRAIL Scale and Kihon checklist preoperatively and at 1 year postoperatively.

Questionnaire	Preoperative	1‐year postoperative	*p*
FRAIL Scale			
Frail	14 (10.0)	7 (5.0)	0.108
Non‐frail	126 (90.0)	133 (95.0)	
Kihon Checklist			
Frail	47 (33.6)	42 (30.0)	0.336
Non‐frail	93 (66.4)	98 (70.0)	
FRAIL Scale			
Frail/pre‐frail	72 (51.4)	44 (31.4)	0.001*
Non‐frail	68 (48.6)	96 (68.6)	
Kihon Checklist			
Frail/pre‐frail	88 (62.9)	80 (57.1)	0.157
Non‐frail	52 (37.1)	60 (42.9)	

* Statistically significant difference (*p* < 0.05). Of the 141 patients, one lacked 1‐year postoperative data for the FRAIL Scale and another for the Kihon Checklist; each analysis was based on 140 patients with complete data.

Table 4 shows the changes in various clinical variables between the preoperative and one‐year postoperative assessments among the 140 patients categorized at baseline as frail/pre‐frail or non‐frail based on the FRAIL scale. In the frail/pre‐frail group, all parameters showed improvement, with Hb, PNI, and PMI improving significantly after surgery. In contrast, although all parameters except BMI improved in the non‐frail group, only PNI showed a statistically significant improvement.

**TABLE 4:** Comparison of changes in clinical variable between frail/pre‐frail and non‐frail groups based on the FRAIL scale preoperatively and at 1 year postoperatively.

Variables	Frail/pre‐frail (*n* = 72)			Non‐frail (*n* = 68)			*p*
	Pre‐ope	1‐year post‐ope	*p*	Pre‐ope	1‐year post‐ope	*p*	
BMI (kg/m^2^)	22.4 ± 3.5	22.7 ± 3.5	0.140	22.6 ± 2.8	22.4 ± 3.1	0.227	0.232
Hb (g/dL)	11.9 ± 2.1	12.8 ± 1.6	0.001*	13.1 ± 1.8	13.5 ± 1.4	0.436	0.089
Alb (g/dL)	3.91 ± 0.4	4.04 ± 0.3	0.061	4.0 ± 0.3	4.1 ± 0.3	0.297	0.845
Prognostic Nutritional Index	46.2 ± 5.3	48.3 ± 4.8	0.001*	48.8 ± 5.0	49.9 ± 6.6	0.012*	0.772
Psoas Muscle Index	4.8 ± 1.4	4.9 ± 1.5	0.019*	5.1 ± 1.7	5.2 ± 1.7	0.093	0.614

Abbreviations: Alb, albumin; BMI, Body Mass Index; Hb, hemoglobin; ope, operation.

* Statistically significant (*p* < 0.05).

While the improvement in Hb was more pronounced in the frail/pre‐frail group compared to the non‐frail group, the difference between the two groups did not reach statistical significance (*p* = 0.089).

## 4 Discussion

This study prospectively evaluated short‐ and long‐term outcomes and changes in frailty status using standardized questionnaire‐based assessments, in CRC patients aged 70 years or older, all of whom underwent MIS, including laparoscopic and robotic procedures. Although OS was significantly worse in the frail group, no significant difference in RFS was detected. Short‐term outcomes were favorable regardless of frailty status. Notably, a significant proportion of patients classified as frail or pre‐frail preoperatively showed improvement in their frailty status at one postoperative year—this is a novel and important finding.

In the frail group, patients were significantly older, had poorer nutritional status, and reduced activities of daily living. While the CCI was comparable between groups, slightly more patients in the frail group had higher scores. Nonetheless, short‐term outcomes, including postoperative complications, did not differ significantly between groups.

Previous studies have reported poorer short‐term outcomes in frail patients undergoing CRC surgery [2, 5]; however, those studies included a substantial proportion of open surgeries. Our findings suggest that, in the current era of MIS, favorable short‐term outcomes can be expected even in frail patients, consistent with prior reports [11, 13, 26]. Nonetheless, some studies have indicated that even MIS does not always ensure favorable outcomes in patients with severely compromised immunity and physiological reserve, [5, 18] requiring cautious interpretation.

This study is novel in that approximately half of the MIS cases were performed using robot‐assisted surgery. Recently, robot‐assisted surgery has been suggested to provide better short‐term outcomes compared to conventional laparoscopic surgery, [27] and it is possible that robotic surgery had some influence on the results of this study. In any case, the effectiveness of MIS, particularly robot‐assisted surgery should be further investigated in frail patients.

Although no significant difference in RFS was observed, OS was significantly worse in the frail group. Previous reviews and meta‐analyses [3, 6, 7] have consistently reported poorer OS and RFS in frail patients after CRC surgery. Considering that frailty itself is associated with poor prognosis in the elderly, [7] these results are unsurprising.

A retrospective study investigating long‐term outcomes in frail patients with Stage I–III colorectal cancer discussed poor RFS being partly attributable to the low tolerability of adjuvant chemotherapy after surgery [3]. Similarly, in this study, adjuvant chemotherapy, particularly oxaliplatin‐based regimens was administered less frequently in the frail group. Moreover, the completion rates of CME and TME, which are both known to improve RFS [28, 29], were lower in the frail group. The frail group was significantly older and had poorer nutritional status, making it clinically challenging to implement these treatments. Although no significant difference in RFS was observed in this study, the divergence of the Kaplan–Meier curves suggests that a significant difference may emerge with a larger sample size. Further large‐scale prospective studies are warranted to validate these findings.

The most noteworthy finding of this study is that even among patients classified as frail or pre‐frail preoperatively, frailty status was significantly improved 1 year postoperatively if they remained recurrence‐free. To date, studies examining longitudinal changes in frailty status among CRC patients have been extremely limited. The recently published GOSAFE study, one of the few addressing postoperative outcomes, demonstrated that preoperative frailty was significantly associated with delayed functional recovery and reduced quality of life 3 and 6 months postoperatively in 625 CRC patients. However, the GOSAFE study focused primarily on short‐term outcomes and did not address long‐term changes in frailty status following MIS [30]. Our study directly evaluated frailty before and 1 year after MIS using standardized tools, providing valuable long‐term follow‐up data rarely reported in the literature.

In the detailed analysis of this study, significant improvements in hemoglobin levels, PNI, and PMI were observed 1 year postoperatively in frail and pre‐frail patients who had undergone curative resection and remained recurrence‐free. These physical indicators respectively reflect different aspects: the PNI represents nutritional status, while PMI reflects muscle mass. In contrast, the FRAIL scale and the Kihon Checklist assess broader domains of functional and physiological vulnerability, including fatigue, physical activity, and weight loss. The overall improvement in these conventional physical indicators likely contributed to the observed improvement in frailty status, suggesting that frailty assessments may capture vulnerabilities not fully reflected by physical indicators alone.

These improvements likely reflect recovery from cancer‐associated cachexia and chronic inflammation following tumor resection [31, 32], providing a theoretical basis for the concept that frailty can be reversible through cancer treatment. This aligns with the emerging concept of “improvement from cancer frailty” proposed in recent reviews [6], and our study offers valuable empirical evidence supporting this view.

Furthermore, as suggested by the GOSAFE study, our findings indicate that surgical resection for CRC may improve overall health status and functional recovery even in older patients. The concept of “improvement from cancer frailty” may thus become an important clinical consideration in oncogeriatric care, extending beyond traditional surgical outcomes.

Several limitations should be considered when interpreting this study. First, it is a single‐center analysis. Patients for whom preoperative surveys could not be conducted due to emergency interventions were not included, and the number of patients who completed the 1‐year postoperative follow‐up was also limited. Therefore, the influence of selection bias cannot be ruled out. Second, as the study did not include an open surgery control group, the specific benefits of MIS could not be fully assessed. Third, although two validated frailty assessment tools were used, psychological and social domains of frailty were not evaluated in detail. Fourth, the completeness of TME, TSME, and CME was assessed intraoperatively by the operating surgeon, which may introduce some degree of subjectivity in the evaluation.

Despite these limitations, two important conclusions can be drawn. First, in elderly frail patients with colorectal cancer, curative MIS did not cause significantly more postoperative complications; however, long‐term survival remained significantly worse than in non‐frail patients. Second, among frail and pre‐frail patients who remained recurrence‐free 1 year postoperatively, frailty status improved significantly. These findings support the proactive application of curative resection in elderly frail patients with CRC, not only from an oncologic standpoint but also due to the potential for improvement in frailty status. Future large‐scale prospective studies incorporating comprehensive frailty assessments are warranted to further elucidate the benefits and limitations of surgical treatment in this population.

## Author Contributions

**Hajime Ushigome:** conceptualization, methodology, investigation, data curation, validation, formal analysis, visualization, writing – original draft, writing – review and editing. **Yushi Yamakawa:** data curation, investigation, validation. **Shunsuke Hayakawa:** conceptualization, methodology. **Akira Kato:** investigation, data curation, validation. **Takuya Suzuki:** investigation, data curation, validation. **Takafumi Sato:** writing – review and editing, writing – original draft. **Hiroyuki Sagawa:** conceptualization, methodology. **Ryo Ogawa:** conceptualization, methodology. **Hiroki Takahashi:** investigation, data curation, validation, supervision. **Shuji Takiguchi:** conceptualization, supervision, methodology.

## Ethics Statement

Author S.T. is an editorial board member of Annals of Gastroenterological Surgery. The study protocol was approved by the institutional review board (Nos. 60‐19‐0150 and 60‐19‐0064). Written informed consent was waived due to the retrospective nature of the study. For the prospective frailty assessment (IRB No. 60‐19‐0064), consent was obtained through an explanatory document and participant agreement via checkbox.

## Conflicts of Interest

The authors declare no conflicts of interest.

## Supporting information


**Table S1:** FRAIL Scale scoring criteria.

**Table S2:** Kihon Checklist scoring criteria.
