# Supplementary material for: Frailty Recovery Following Minimally Invasive Surgery: An Emerging Perspective on Surgical Benefits in Elderly Colorectal Cancer Patients
Source: Ann Gastroenterol Surg. 2025 Aug 3;10(1):137–44. doi: 10.1002/ags3.70070 (PMC12757148; doi:10.1002/ags3.70070)
Supplement: Supplementary file 1 — Table S1: FRAIL Scale scoring criteria. [file AGS3-10-137-s002.docx]

Supplemental Table. **FRAIL Scale Questionnaire and Scoring criteria**

| Item | Question | Scoring |
| --- | --- | --- |
| Fatigue | Do you feel tired most of the time? | Yes = 1 / No = 0 |
| Resistance | Can you climb one flight of stairs without rest? | Yes = 0 / No = 1 |
| Ambulation | Can you walk one block unaided? | Yes = 0 / No = 1 |
| Illnesses | Do you have five or more illnesses? | Yes = 1 / No = 0 |
| Loss of Weight | Have you lost more than 5% of your weight in the last year? | Yes = 1 / No = 0 |
| Total Score |  | 1-2: Pre-frail 3-5: Frail |
