# Supplementary material for: Frailty Recovery Following Minimally Invasive Surgery: An Emerging Perspective on Surgical Benefits in Elderly Colorectal Cancer Patients
Source: Ann Gastroenterol Surg. 2025 Aug 3;10(1):137–44. doi: 10.1002/ags3.70070 (PMC12757148; doi:10.1002/ags3.70070)
Supplement: Supplementary file 2 — Table S2: Kihon Checklist scoring criteria. [file AGS3-10-137-s001.docx]

Supplemental Table. 　**Kihon Checklist Questionnaire and Scoring criteria**

| No. | Item | Question | Scoring |
| --- | --- | --- | --- |
| 1 | ADL | Do you go out by bus or train by yourself? | Yes = 0 / No = 1 |
| 2 | ADL | Do you go shopping to buy daily necessities by yourself? | Yes = 0 / No = 1 |
| 3 | ADL | Do you manage your own deposits and savings at the bank? | Yes = 0 / No = 1 |
| 4 | ADL | Do you sometimes visit your friends? | Yes = 0 / No = 1 |
| 5 | ADL | Do you turn to your family or friends for advice? | Yes = 0 / No = 1 |
| 6 | Physical Function | Do you normally climb stairs without using handrail or wall for support? | Yes = 0 / No = 1 |
| 7 | Physical Function | Do you normally stand up from a chair without any aids? | Yes = 0 / No = 1 |
| 8 | Physical Function | Do you normally walk continuously for 15 minutes? | Yes = 0 / No = 1 |
| 9 | Physical Function | Have you experienced a fall in the past year? | Yes = 1 / No = 0 |
| 10 | Physical Function | Do you have a fear of falling while walking? | Yes = 1 / No = 0 |
| 11 | Nutritional Status | Have you lost 2 kg or more in the past 6 months? | Yes = 1 / No = 0 |
| 12 | Nutritional Status | Is your BMI less than 18.5 kg/m2? | Yes = 1 / No = 0 |
| 13 | Oral Function | Do you have any difficulties eating tough foods compared to 6 months ago? | Yes = 1 / No = 0 |
| 14 | Oral Function | Have you choked on your tea or soup recently? | Yes = 1 / No = 0 |
| 15 | Oral Function | Do you often experience having a dry mouth? | Yes = 1 / No = 0 |
| 16 | Social Engagement / Social Withdrawal | Do you go out at least once a week? | Yes = 0 / No = 1 |
| 17 | Social Engagement / Social Withdrawal | Do you go out less frequently compared to last year? | Yes = 1 / No = 0 |
| 18 | Cognitive Function | Do your family or friends point out your memory loss? | Yes = 1 / No = 0 |
| 19 | Cognitive Function | Do you make a call by looking up phone numbers? | Yes = 0 / No = 1 |
| 20 | Cognitive Function | Do you find yourself not knowing today’s date? | Yes = 1 / No = 0 |
| 21 | Depression Risk / Depressive Symptoms | In the last 2 weeks, have you felt a lack of fulfilment in your daily life? | Yes = 1 / No = 0 |
| 22 | Depression Risk / Depressive Symptoms | In the last 2 weeks, have you felt a lack of joy when doing the things you used to enjoy? | Yes = 1 / No = 0 |
| 23 | Depression Risk / Depressive Symptoms | In the last 2 weeks, have you felt difficulty in doing what you could do easily before? | Yes = 1 / No = 0 |
| 24 | Depression Risk / Depressive Symptoms | In the last 2 weeks, have you felt helpless? | Yes = 1 / No = 0 |
| 25 | Depression Risk / Depressive Symptoms | In the last 2 weeks, have you felt tired without a reason? | Yes = 1 / No = 0 |
|  |  | Total Score 4-7: Pre-frail, 7< Frail | |
